# A Population-Based Study on the Association between Periodontal Disease and Major Lifestyle-Related Comorbidities in South Korea: An Elderly Cohort Study from 2002–2015

**DOI:** 10.3390/medicina56110575

**Published:** 2020-10-29

**Authors:** Jae-Hong Lee, Seong-Nyum Jeong

**Affiliations:** Department of Periodontology, Daejeon Dental Hospital, Institute of Wonkwang Dental Research, College of Dentistry, Wonkwang University, Daejeon 35233, Korea; seongnyum@wku.ac.kr

**Keywords:** aged, comorbidity, periodontal diseases, periodontitis, risk factors

## Abstract

This study determined the association between periodontal disease (PD) and major lifestyle-related comorbidities (LCs) using the database of the nationwide population-based National Health Insurance Service–Elderly Cohort 2002–2015. A nationwide representative sample comprising 558,147 participants, aged 60 years, was analyzed. Univariate and multivariate logistic regression analyses adjusted for sociodemographic and economic factors (sex, age, household income, insurance status, health status, and living area) and major LCs (hypertension, diabetes mellitus, rheumatoid arthritis, osteoporosis, cerebral infarction, angina pectoris, myocardial infarction, erectile dysfunction, lipoprotein disorder, and obesity) were used to determine the association between PD and major LCs. Elderly participants with PD had a higher risk of major LCs (hypertension: odds ratio (OR) = 1.40, diabetes mellitus: OR = 1.22, rheumatoid arthritis: OR = 1.16, osteoporosis: OR = 1.37, erectile dysfunction: OR = 1.73, lipoprotein disorder: OR = 1.50, and obesity: OR = 1.59). Our longitudinal cohort study provided evidence that PD was significantly associated with major LCs in elderly participants. In particular, the association between PD and erectile dysfunction had the highest OR in the multivariate analyses.

## 1. Introduction

Periodontal disease (PD) is a highly prevalent and multifactorial chronic inflammatory oral disorder with a complex etiology involving both bacterial and environmental components [1,2]. PD causes destruction of tooth-supporting tissues, including the alveolar bone, cementum, and periodontal ligament, and finally leads to tooth loss, significantly reducing masticatory function and quality of life [3].

As a result of rapidly aging population, urbanization, and industrialization, many chronic inflammatory and infectious diseases have become more prevalent worldwide [4]. Lifestyle-related comorbidities (LCs) or diseases, such as cardiovascular disease, hypertension, obesity, and diabetes mellitus, significantly deteriorate the quality of life and are a major cause of morbidity and mortality in elderly individuals [5].

PD and LCs are among the most common non-communicable diseases and are closely related to factors such as poor dietary habits and smoking, and both diseases are regarded as risk factors or indicators that affect the outcome of one another [6,7]. In recent years, epidemiological and pathological studies have documented the bidirectional relationship between PD and LCs and have described several similar etiological and pathophysiological pathways between the conditions [6,7]. In particular, remarkable associations have been reported between LCs and gram-negative periodontal pathogens, including *Aggregatibacter actinomycetemcomitans* and *Porphyromonas gingivalis* [8,9].

Substantial evidence has shown that bacteria, endotoxins, and serum and salivary mediators associated with PD can directly or indirectly lead to bacteremia, systemic inflammation, and endothelial dysfunction [10,11,12,13]. In particular, chronic and severe PD has a significantly increased risk of developing LCs after adjusting for many of the confounding risk factors [14]. Nevertheless, the underlying plausible mechanisms linking PD with LCs are not fully understood, and there is limited evidence available from controlled trials. In the past decades, various cohort studies have confirmed the association between periodontal disease and LCs, but large cohort studies involving elderly people aged 65 years or older are still insufficient to clarify the causative relationship. Therefore, we designed a retrospective cohort study using the database of the nationwide population-based National Health Insurance Service–Elderly Cohort (NHIS–EC) to investigate the association between PD and major LCs among elderly participants in South Korea.

## 2. Materials and Methods

### 2.1. Study Design and Data Collection

This study conformed to the Strengthening the Reporting of Observational Studies in Epidemiology (STROBE) guidelines for reporting observational cohort studies (www.strobe-statement.org) and was approved by the Institutional Review Board of Daejeon Dental Hospital, Wonkwang University (approval no. W1908/003-001). This retrospective elderly cohort study used data collected over 14 years (2002–2015) from the National Health Insurance Service Sharing Service (NHISS, https://nhiss.nhis.or.kr). Four categories of data were extracted from the NHIS–EC database: sociodemographic and economic information, insurance status, health checkup examination, and medical and dental records. Details of the NHIS cohort profile have been described in a reference study [15].

### 2.2. Study Participants

The nationwide database from the South Korean mandatory National Health Insurance Program consists of a representative sample of 558,147 participants, with 10% of elderly people aged 60 years and above. We identified participants who had received a diagnosis of acute or chronic PD based on the following International Classification of Diseases, 11th Revision (ICD-11) diagnostic codes: acute periodontitis (DA0C.0), aggressive periodontitis (DA0C.1), periodontosis (DA0C.2), necrotizing PD (DA0C.3), abscess of the periodontium (DA0C.4), linear gingival erythema (DA0C.5), other specified PD (DA0C.Y), and unspecified PD (DA0C.Z). PD was diagnosed clinically and radiographically on the basis of probing depth, clinical attachment level, and bone loss in accordance with the guidelines of the American Academy of Periodontology [16]. In addition, only participants with these codes who were insured at least three times between 2002 and 2005 were included to increase diagnostic accuracy.

All participants were followed-up from the entry date of the cohort until the date of LC diagnosis (according to the following ICD-11 diagnostic codes: hypertension (BA00), diabetes mellitus (5A10–5A14), rheumatoid arthritis (FA20), osteoporosis (FB83.1), cerebral infarction (8B11), angina pectoris (BA40), acute and subsequent myocardial infarction (BA41–BA42), erectile dysfunction (ED) (HA01.1), lipoprotein disorder (OC80.00, 5C81Z, 5C8Y, and 5C8Z), and obesity (5B81)), migration, mortality, or the end of the study period.

### 2.3. Covariates

The covariates included in the analyses were sex (male and female), age (six groups: age 60–64, 65–69, 70–74, 75–79, 80–84, and ≥85 years), household income (five groups: classified according to the insurance fee imposed on each household, with Medical Aid Program (MAP) beneficiaries in the first quintile), insurance status (three groups: those classified as MAP and NHIS (employees and self-employed)), health status (two groups: those classified as healthy and those with major and minor disabilities according to the Handicapped Welfare Law), and living area (three groups: those living in Seoul (≥10 million residents), metropolitan areas (≥1 million residents), and other areas (<1 million residents)).

### 2.4. Statistical Analysis

The chi-squared test was used to assess differences in categorical variables between the PD and periodontally healthy participants. Univariate and multivariate logistic regression analyses adjusted for sociodemographic and economic factors (sex, age, household income, insurance status, health status, and living area) and LCs (hypertension, diabetes mellitus, rheumatoid arthritis, osteoporosis, cerebral infarction, angina pectoris, myocardial infarction, erectile dysfunction, lipoprotein disorder, and obesity) were used to examine the risk factors for PD, and the results were presented as odds ratios (ORs) and 95% confidence intervals (CIs). All statistical analyses were performed using SAS software (version 9.4, SAS Institute, Cary, NC, USA), and a *p* value <0.05 was considered to be statistically significant.

## 3. Results

### 3.1. Sociodemographic and Economic Factors Associated with Periodontal Disease

The sociodemographic and economic factors of the PD and periodontally healthy participants are summarized in Table 1. Among the 558,147 South Korean participants originally included in 2002–2015, 149,785 participants were diagnosed with PD. Those aged 60–64 years (*n* = 75,716) accounted for 50.5% of the surveyed participants. Furthermore, 51,248 (34.2%) of the participants were in the fifth quintile of household income, 83,003 (55.4%) were employed, 149,302 (99.7%) had a healthy health status, and 78,957 (52.7%) lived in areas with <1,000,000 residents.

### 3.2. Lifestyle-Related Comorbidities Associated with Periodontal Disease

A higher prevalence of all surveyed LCs was noted in the PD participants compared to the periodontally healthy participants (*p* < 0.001) (Table 2). The most prevalent LC among participants was hypertension (*n* = 293,246; 52.5%), followed by osteoporosis (*n* = 187,904; 33.7%), lipoprotein disorder (*n* = 186,083; 33.3%), diabetes mellitus (*n* = 176,914; 31.7%), angina pectoris (*n* = 99,747; 17.9%), cerebral infarction (*n* = 96,873; 17.4%), rheumatoid arthritis (*n* = 73,609; 13.2%), acute myocardial infarction (*n* = 15,378; 2.8%), erectile dysfunction (*n* = 2,557; 0.5%), subsequent myocardial infarction (*n* = 876; 0.2%), and obesity (*n* = 667; 0.1%).

**Figure 1:**
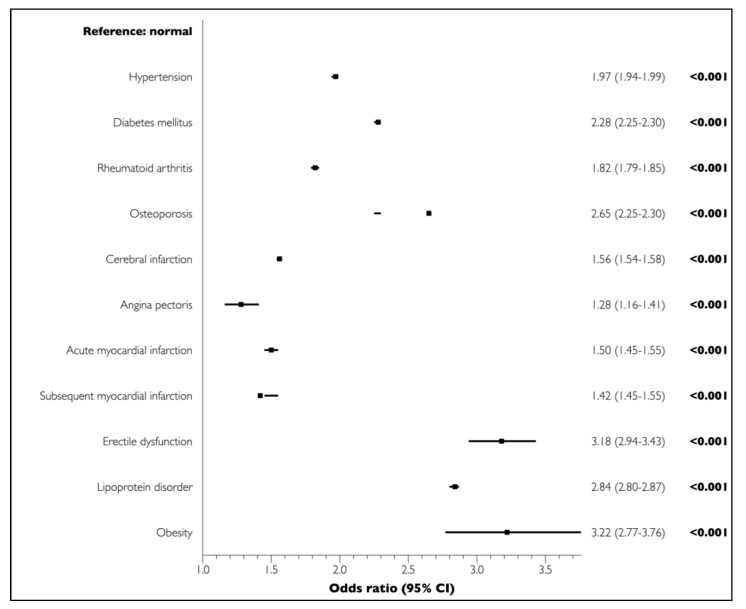
Associations of lifestyle-related comorbidities (LCs) with PD in elderly participants in the univariate analysis.

**Figure 2:**
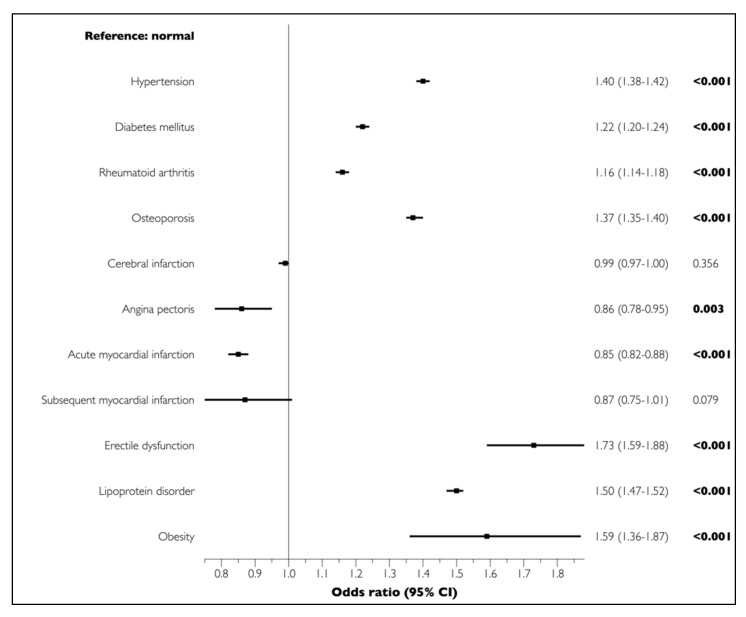
Associations of LCs with PD in elderly participants in the multivariate analysis. Boldface denotes statistical significance (*p* < 0.05). Multivariate logistic regression analyses adjusted for sociodemographic and economic factors (sex, age, household income, insurance status, health status, and living area) and LCs (hypertension, diabetes mellitus, rheumatoid arthritis, osteoporosis, cerebral infarction, angina pectoris, myocardial infarction, erectile dysfunction, lipoprotein disorder, and obesity).

The univariate analysis demonstrated significant associations between all LCs and PD (*p* < 0.001) (Figure 1). The multivariate regression analysis adjusted for sociodemographic and economic factors and LCs showed that cerebral infarction (OR = 0.99, 95% CI = 0.97–1.00, *p* = 0.356), angina pectoris (OR = 0.86, 95% CI = 0.78–0.95, *p* = 0.003), acute myocardial infarction (OR = 0.85, 95% CI = 0.82–0.88, *p* < 0.001), and chronic myocardial infarction (OR = 0.87, 95% CI = 0.75–1.01, *p* = 0.079) were not positively associated with PD. In contrast, all other LCs were significantly and positively associated with PD (Figure 2).

## 4. Discussion

This long-term retrospective cohort study indicated that hypertension, diabetes mellitus, rheumatoid arthritis, osteoporosis, erectile dysfunction, lipoprotein disorder, and obesity were significantly and positively associated with PD when adjusted for sociodemographic and economic factors and major LCs. These findings are consistent with the results obtained in previous cohort studies involving young and middle-aged adults [17,18].

Several possible mechanisms may be considered in establishing the relationship between PD and LCs [19,20,21]. First, inflammation plays an important role in the pathogenesis of local and systemic conditions. However, inflammatory mediators causing certain LCs are not well-defined. Systemic inflammation, as observed by an increase in the serum levels of C-reactive protein, and other biomarkers resulting from PD may be considered as one pathway by which this oral disease increases the risk of various LCs. Second, pathological oral bacteria may induce traumatic injury and cause irritation of the epithelium and mucosa, thereby playing a role in the subsequent progression of LCs. Other mechanisms, including a compromised immune system and inflammatory byproducts of periodontal pathogens, have also been proposed as possible associations.

Chronic and localized oral inflammation induces systemic inflammation and endothelial dysfunction, and is also intimately associated with ED. A number of potential risk factors, including increasing age, diabetes mellitus, hyperlipidemia, hypertension, and smoking, have been suggested to be important contributors to the development and progression of both PD and ED [22,23]. In the present study, the association between PD and ED showed the highest OR (1.73, 95% CI = 1.59–1.88, *p* < 0.001) in the multivariate analysis. A systematic review, based on cross-sectional and randomized controlled trials, reported a positive association (OR = 1.53–3.35) between PD and ED, and a recent meta-analysis also confirmed that PD increased the risk of occurrence of ED (OR = 2.85, 95% CI = 1.83–4.46, *p* < 0.01) [24,25]. Moreover, it was reported that the risk for ED prevalence was 3.07 times (95% CI = 1.87–5.05, *p* < 0.01) higher in Asian men [25].

In the present study, obesity, lipoprotein disorder, and hypertension had the second (OR = 1.59, 95% CI = 1.36–1.87, *p* < 0.001), third (OR = 1.50, 95% CI = 1.47–1.52, *p* < 0.001), and fourth (OR = 1.40, 95% CI = 1.38–1.42, *p* < 0.001) highest ORs of all surveyed LCs, respectively. Several epidemiological, experimental, and clinical studies have investigated and explored the potential role of obesity and hypertension in the development and progression of PD [26,27,28]. In one of the first animal experiments, obese rats showed the highest degree of alveolar bone loss, and obese hypertensive rats showed the most severe periodontal destruction [26]. A recent systematic review showed that hypertension was associated with PD (OR = 1.50, 95% CI = 1.27–1.78, *p* < 0.001), especially severe PD (OR = 1.64, 95% CI = 1.23–2.19, *p* < 0.001) [29]. A systematic review and meta-analysis reported a positive association between PD and obesity (OR = 1.35, 95% CI = 1.23–1.47, *p* < 0.005), validating the role of obesity in the development of PD [27]. Another recent literature review suggested that obesity and metabolic diseases (such as dyslipidemia, hypertension, and dysglycemia) may be risk factors for the development and progression of PD [30,31,32].

The genetic basis of the association between PD and lipoprotein disorder is unclear and probably multifactorial; however, both diseases may be risk factors for obesity and poorly controlled diabetes mellitus [33]. Chronic PD produces endotoxins, which increases the serum levels of low-density lipoprotein cholesterol and has a negative effect on glycemic control [5,34]. Circulating serum levels of pro-inflammatory mediators, such as interleukin (IL)-1β, IL-6, tumor necrosis factor-α, C-reactive protein, and matrix metalloproteinases, are associated with both PD and lipoprotein disorders in elderly individuals [35,36].

In the present study, participants with PD exhibited an increased risk of osteoporosis (OR = 1.37, 95% CI = 1.35–1.40, *p* < 0.001). PD and osteoporosis have common lifestyle and biological risk factors, namely, old age, poor nutritional status, immunological diseases, and smoking [37]. Chronic and low-grade systemic inflammation may induce bone loss and reduce bone density, increasing the risk of osteoporotic fractures and promoting the progression of PD and tooth loss [38]. Recent systematic reviews have reported a probable association between PD and osteoporosis [39,40]. In particular, one systematic review found that chronic and severe PD was strongly associated with osteoporosis in postmenopausal women. However, further clinical studies are necessary to confirm this hypothesis [41].

Several studies have supported a biologically plausible association between PD and rheumatoid arthritis [42,43]. Both disorders are highly prevalent inflammatory and multifactorial diseases that share common risk factors and risk indicators. *P. gingivalis* lipopolysaccharide, which is involved in the development and progression of PD, was shown to induce the expression of pro-inflammatory cytokines and chemokines, thereby increasing the severity of rheumatoid arthritis [44]. This suggests that rheumatoid arthritis may be caused by dysbiosis of the periodontal microbiota [44,45]. In a recent study on the genetics of PD and rheumatoid arthritis, an allele of the interferon-gamma gene was identified as a significant disease-specific marker common to both diseases [43].

A recent umbrella review (review of systematic reviews or meta-analyses) reported that the most frequent correlation was found for PD with cardiovascular diseases [46]. As cardiovascular diseases, especially acute myocardial infarction, are associated with high mortality, morbidity, and serious sequelae in the elderly population, a significant number of elderly participants with these diseases would have been excluded from the NHIS–Elderly Cohort database. It was suspected that PD patients with severe and progressive cardiovascular disease were unlikely to visit dental clinics to be diagnosed and treated with PD, resulting in a low and insignificant negative correlation [47].

There are inherent limitations in conducting this retrospective observational cohort study based on the NHIS–EC database. First, many previous studies have clearly reported that smoking promotes the development and progression of PD and LCs [48,49,50]. Thus, a major limitation of the current study is that information on the smoking status of the participants was not included. Second, this retrospective cohort study did not assess dental records such as periodontal probing charts and radiographs, which limited the ability to diagnose the severity or extent of PD. In addition, the fact that the recently updated classification system for PD was not applied to the current cohort database can be considered as one of the limitations.

## 5. Conclusions

Our longitudinal cohort study suggests that PD is associated with a higher risk of most major LCs in elderly people. In particular, among the investigated LCs, erectile dysfunction seems to have a significant association with PD. However, no definitive conclusions could be drawn regarding the causative relationship between the underlying biological mechanisms of PD and LCs. Therefore, further large-scale and well-designed controlled clinical trials are needed to find conclusive evidence.

## Figures and Tables

**Table 1:** Sociodemographic and economic factors associated with periodontal disease (PD).

Variables	PD Participants		Periodontally Healthy Participants		*p* Value
	*n*	%	*n*	%	
**Total**	149,785	100.0	408,362	100.0	
**Sex**					
Male	65,817	43.9	167,765	40.3	<0.001
Female	83,968	56.1	243,597	59.7	
**Age group (years)**					
60–64	75,716	50.5	120,400	29.5	<0.001
65–69	44,322	29.6	103,039	25.2	
70–74	20,179	13.5	77,478	19.0	
75–79	7234	4.8	53,983	13.2	
80–84	1970	1.3	33,245	8.1	
≥85	364	0.2	20,217	5.0	
**Household income ^1^**					
First quintile	26,179	17.5	104,610	25.6	<0.001
Second quintile	17,264	11.5	53,158	13.0	
Third quintile	21,944	14.7	59,686	14.6	
Fourth quintile	33,150	22.1	81,155	19.9	
Fifth quintile	51,248	34.2	109,753	26.9	
**Insurance status**					
MAP beneficiary	6057	4.0	39,369	9.6	<0.001
NHIS, employed	83,003	55.4	194,955	47.7	
NHIS, self-employed	60,725	40.5	174,038	42.6	
**Health status ^2^**					
Healthy	149,302	99.7	405,175	99.2	<0.001
Disabled	483	0.3	3187	0.8	
**Living area ^3^**					
Seoul	33,714	22.5	65,878	16.1	<0.001
Metropolitan area	37,114	24.8	85,758	21.0	
Other areas	78,957	52.7	256,726	62.9	

PD, periodontal disease; MAP, Medical Aid Program; NHIS, National Health Insurance Service. ^1^ Quintiles based on the insurance fee imposed on each household (with MAP beneficiaries in the first quintile). ^2^ Classification based on the Handicapped Welfare Law in South Korea. ^3^ Classification based on residency in Seoul (≥10,000,000 residents), metropolitan areas (≥1,000,000 residents), or other areas (<1,000,000 residents).

**Table 2:** Sociodemographic and economic factors associated with PD.

Variables	PD Participants		Periodontally Healthy Participants		*p* Value
	*n*	%	*n*	%	
**Total**	149,785	100.0	408,362	100.0	
Hypertension	104,257	69.6	188,989	46.3	<0.001
Diabetes mellitus	67,946	45.4	108,968	26.7	<0.001
Rheumatoid arthritis	27,971	18.7	45,638	11.2	<0.001
Osteoporosis	67,623	45.1	120,281	29.5	<0.001
Cerebral infarction	33,467	22.3	63,406	15.5	<0.001
Angina pectoris	39,547	26.4	60,200	14.7	<0.001
Myocardial infarction					
Acute	5433	3.6	9945	2.4	<0.001
Subsequent	300	0.2	576	0.1	<0.001
Erectile dysfunction	1373	0.9	1184	0.3	<0.001
Lipoprotein disorder	76,465	51.0	109,618	26.8	<0.001
Obesity	362	0.2	305	0.1	<0.001
